# Prediction of dementia using CT imaging in stroke (PRODUCTS)

**DOI:** 10.1177/23969873251325076

**Published:** 2025-03-13

**Authors:** Melanie Hafdi, Martin Taylor-Rowan, Bogna Drozdowska, Emma Elliott, Lucy McGuire, Edo Richard, Terence J Quinn

**Affiliations:** 1 Department of Neurology, Amsterdam UMC, University of Amsterdam, Amsterdam, The Netherlands; 2 Institute of Health and Wellbeing, University of Glasgow, Glasgow, UK; 3 Cumming School of Medicine, University of Calgary, Calgary, Canada; 4 National Institute for Health and Care Research (NIHR) Applied Research Collaboration Greater Manchester, School of Health Sciences, The University of Manchester, Manchester, UK; 5 Institute of Cardiovascular and Metabolic Sciences, University of Glasgow, Glasgow, UK; 6 Department of Neurology, Donders Institute for Brain, Behaviour and Cognition, Radboud University Medical Centre, Nijmegen, The Netherlands; 7 Department of Public & Occupational Health, University of Amsterdam, Amsterdam, The Netherlands

**Keywords:** Stroke imaging, post-stroke dementia, CT imaging, brain frailty

## Abstract

**Introduction:**

A better understanding of who will develop dementia can inform patient care. Although MRI offers prognostic insights, access is limited globally, whereas CT-imaging is readily available in acute stroke. We explored the prognostic utility of acute CT-imaging for predicting dementia.

**Patients and methods:**

We included stroke or transient ischaemic attack (TIA) survivors from participating stroke centres in Scotland. Acute CT-scans were rated using ordinal scales for neurodegenerative and cerebrovascular changes (old infarcts, white matter lesions (WMLs), medial temporal lobe atrophy (MTA), and global atrophy (GA)) and combined together to a ‘brain-frailty’ score. Dementia status was established at 18-months following stroke or TIA.

**Results:**

Among 195 participants, 33% had dementia after 3 years of follow-up. High brain-frailty score (⩾2/4) correlated with higher risk of dementia (HR (95% CI) 6.02 (1.89–19.21)). As individual predictor, severe MTA was most strongly associated with dementia (adjusted HR (95% CI) 2.09 (1.07–4.08)). Other predictors associated with dementia included older age, higher prestroke morbidity (mRS), WMLs, and GA. Integrated in a prediction model with clinical parameters, prestroke mRS, cardiovascular disease, GA, MTA and Abbreviated-Mental-Test were the strongest predictors of dementia (c-statistic: 0.77).

**Discussion and conclusion:**

Increased brain-frailty, and its individual components (WMLs, MTA, and GA) are associated with a higher risk of dementia in participants with stroke. Combining clinical and brain-frailty parameters created a moderate dementia prediction model but added little value over clinical parameters in combination with cognitive testing. CT-based brain-frailty may provide better prognostic insights when cognitive testing isn’t feasible and for identifying highest-risk individuals for dementia prevention trials to increase trial efficiency.

## Graphical abstract

**Graphical abstract:**
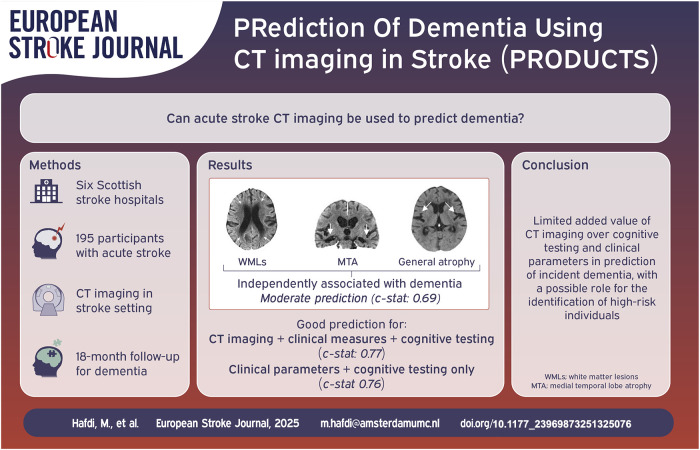

## Introduction

Stroke, as a leading cause of disability worldwide, often results in not only acute neurological deficits but also long-term cognitive impairment.^1,2^ Up to 30% of patients who suffered stroke develop dementia in the first 5 years after diagnosis and cognitive deficits are present in over 70% of stroke survivors.^3,4^ Understanding who is at risk of developing dementia allows for advance care planning, timely interventions, and efficient resource allocation, potentially mitigating further disease progression and improving patient outcomes. Various magnetic resonance imaging (MRI) biomarkers for dementia prediction have been described, including measures assessed in acute stroke imaging, but they have had limited clinical traction, potentially due to burdensome imaging protocols and limited availability of the equipment and software required.^5,6^ Stroke and dementia incidence is expected to increase dramatically in low- and middle income countries (LMIC) where MRI scanners are not routinely available, which further stresses the necessity for a cheaper and more widely available test.^7,8^

Computed tomography (CT) imaging, a widely accessible and routinely utilized diagnostic modality in acute stroke management, presents a promising avenue for investigating potential imaging biomarkers that may portend future dementia risk.^9^ Plain CT scanning at time of stroke allows for assessment of structural markers that may suggest concomitant neurodegeneration for example, global or regional atrophy and also markers of underlying cerebrovascular pathology including white matter lesions or (lacunar) stroke. Differing CT features can be combined to give an overall assessment, often referred to as ‘brain health’ or ‘brain frailty’.

Though CT scan is the most commonly used imaging modality, it is under-researched as a prognostic tool for dementia.^6^ The recent European Stroke Organisation and European Academy Neurology joint guidelines on post-stroke cognitive impairment reported on the association of several findings on acute imaging, for example, white matter lesions and silent infarctions, with concomitant post-stroke dementia but concluded that there is more evidence needed for the predictive value of acute CT-brain imaging for cognitive outcomes.^9^

Our aim was to investigate the association of imaging markers derived from real world acute stroke CT brain scans with dementia. Our primary aim was to calculate a score for brain frailty and investigate its prognostic properties for predicting dementia. As a secondary aim, we assessed the value of this brain frailty measure for predicting dementia both when added to clinical and demographics variables and in comparison to these variables with no additional imaging data.

By elucidating the prognostic significance of CT findings in the acute stroke setting, this research endeavours to help refine risk stratification strategies and inform possible future targeted therapeutic interventions aimed at mitigating dementia risk in stroke survivors.

## Methods

This study is reported according to the Transparent Reporting of a multivariable prediction model for Individual Prognosis Or Diagnosis (TRIPOD) statement.^10^ This study is a substudy of the ‘Assessing Psychological Problems In Stroke: A Longitudinal Evaluation’ project (APPLE; research registry ID: 1018) – a multicentre, prospective observational cohort study embedded within the UK National Health Service. Comprehensive details on the study are available in the study protocol.^11^ This substudy was restricted to data from those sites with CT brain images available for analysis. Ethical approval was obtained for all participating sites (REC number: 16/SS/0105).

### Study design and participants

The APPLE project recruited participants with a clinical diagnosis of stroke or transient ischaemic attack (TIA) via six participating Scottish hospitals from November 2016 to February 2019 and followed them up with neurocognitive assessments. The only exclusion criteria were inability to provide consent, no spoken English (pre-stroke) and prisoners. Patients unable to provide consent at baseline due to severe aphasia could still be included if a suitable proxy provided assent. Demographical and clinical details (e.g. National Institute of Health Stroke Scale (NIHSS) score) were from clinical assessment or extracted from case notes. Cardiovascular disease was self-reported and cross-checked with medical records and was defined as a composite of ischaemic heart disease, coronary artery interventions, myocardial infarction, and peripheral vascular disease. Information about cognitive and functional status such as prestroke modified Rankin Scale (mRS), and Abbreviated Mental Test (AMT-10 score; a brief cognitive screening instrument^11^) was collected during the baseline assessment. The demographic and clinical characteristics of the full APPLE cohort, previously published^11^ and compared to the Scottish Stroke Care Audit,^12^ showed that while the cohort was slightly younger than the unselected national data (70 years vs 73 years respectively), it was otherwise comparable.

### CT assessment

All participants underwent acute CT brain imaging following admission to the hospital for stroke. We evaluated CT scans for neurovascular and -degenerative changes, including old infarction (any type of old infarction damage visible on CT, irrespective of the specific stroke subtype), white matter lesions (WMLs; Fazekas et al.^13^ scale), medial temporal lobe atrophy (MTA; Scheltens et al.^14^ scale), and atrophy (Wahlund et al.^15^ scale). We established an overall ‘brain frailty’ score^16^ for each participant by combining the presence of old infarcts, severe WMLs (score of >2 on either component of Fazekas scale; range 0–3), and severe medial-temporal or global atrophy (score of ⩾2 on left or right Schelten’s scale – ⩾3 for those ⩾75 years old; range 0–4 – or ⩾2.5 on any component of Wahlund scale; range 0–3). One point was assigned for each component with higher scores indicating more brain frailty (range 0–4).

CT scans were rated by two trained researchers (LM, MH) who were blinded to clinical and cognitive outcome data. To ascertain internal validity, we double scored 10% of our included scans (addressing intra-observer variability) and scored a further 10% by an independent rater (MT-R; addressing inter-observer variability) at an early stage of assessment. If the variability of scoring was too high (i.e. Cohen’s kappa < 0.60), we planned to re-train scoring techniques and reassess scored scans.

### Assessment of dementia

The study comprised a maximum of seven cognitive assessments: a short baseline assessment during admission to the acute stroke unit; (optional) semi-structured clinical interview within first month; 1 month follow-up with short screening tests; then 6, 12, 18-month follow-ups with multi-domain testing based on the National Institute of Neurological Disorders and Stroke-Canadian Stroke Network vascular cognitive impairment harmonization standards and with an optional clinical diagnostic assessment.^17^ A comprehensive overview of the cognitive testing protocol, including utilized cognitive tests has been described previously.^11^ Briefly, a standardized cognitive test battery was implemented across all participating sites, following an iterative approach tailored to each participant’s capacity to engage with the testing process. To be included in the cognitive outcome adjudication every participant had at least two follow up visits with multidomain cognitive assessment. Home assessment or telephone assessment was possible if required. Two independent assessors (medical doctor and psychologist) reviewed all primary and secondary care clinical case notes (up to a 3 year follow-up timeframe) and in-study data (up to 18-months). A consensus dementia diagnosis was established by integrating information from cognitive tests, informant-based questionnaires, and all available clinical records. The date of dementia for the Cox regression models was defined as either the date of diagnosis recorded in the participant’s clinical file or the date of a cognitive testing session (conducted at 6-month intervals) where dementia was first identified. During the time period of the study, features such as atrophy and white matter disease were not routinely reported for scans performed for acute stroke indications. Thus while the consensus review panel may have had access to CT scan reports, the final classification would not have been based on brain frailty or its components, minimizing incorporation bias. A consensus diagnosis on all-cause dementia (classified as major neurocognitive disorder not present, possible, or probable) was assigned according to the Diagnostic and Statistical Manual (DSM5) criteria.^18^ As a quality check, all dementia diagnoses were re-evaluated by an independent adjudicator (TQ; geriatrician). In this cohort, participants with pre-stroke dementia were not excluded as the CT markers under study are not specific to post-stroke dementia alone.

### Statistical analyses

As a first step, we assessed the strength of association for individual and combined CT parameters with dementia (possible and probable combined) using a cox-proportional hazard model. In model 2, we corrected for age and sex, in model 3 we additionally adjusted for history of cardiovascular disease and prestroke mRS, and in model 4 additionally for previous stroke and National Institutes of Health Stroke Scale score (NIHSS). We checked robustness of our findings by adding a Fine and Grey model to evaluate the competing risk of death. We performed predefined sensitivity analyses on individual components of the brain frailty score and the effect of increasing brain frailty on dementia outcomes. We assessed the discriminative properties of individual CT parameters by calculating and plotting the area under the receiver operating characteristic curve (AUC).

To develop a risk prediction model, we included relevant clinical and demographic variables known to be associated with (post-stroke) dementia^19,20^ and available in the study data-set (i.e. age, sex, history of cardiovascular disease and stroke (self-reported), prestroke mRS, stroke severity (NIHSS) and AMT-10 score) and individual brain frailty components. We used predictive mean matching multiple imputation for missing values. We applied multivariate Cox proportional hazards analysis and used backward stepwise selection for variable selection as this has been described to render reliable predictors.^21^ Variable exclusion was performed using a 10% significance level as a stopping criterion. We calculated discrimination indexes for the prediction model using concordance (c)-statistics, with values lower than 0.5 indicating an unacceptable model, 0.5–0.6 a low -, 0.61–0.8 a moderate -, 0.81–0.9 a good - and 0.91–1 a very good discriminative model.^22,23^ We checked robustness by subsequently analysing a model consisting of only brain frailty and only clinical components and by calculating c-statistics for unselected models incorporating all variables.

All analyses were carried out using R statistical software, version 4.2.1.

## Results

### Descriptives

Table 1 provides baseline characteristics. Out of 332 participants who participated in the APPLE study, 195 had raw CT images available and were assessed for cognitive outcomes over 18 months of follow-up, of whom 64 (33%) had a dementia diagnosis after 3 years of follow-up. Complete follow-up data for dementia was obtained for all assessed participants. The mean age of the population was 70 ± 12 years and 52% were male. Vascular risk factors (i.e. atrial fibrillation, diabetes, heart failure, hypertension, dyslipidaemia and self-reported previous stroke) did not differ between participants with or without dementia. Participants that with dementia were older (mean age 75 ± 10 years vs 67 ± 13 years) and had a higher prestroke mRS (1.99 ± 1.27 vs 0.99 ± 1.16). CT characteristics are listed in Table 2. Stroke aetiology (haemorrhagic vs ischaemic) and old infarct damage did not differ between participants, whereas white matter lesions, medial temporal lobe atrophy and general atrophy were all higher in participants with dementia (Table 2). The mean brain frailty score was higher in participants with dementia when compared to those without (3.24 ± 0.89 vs 2.37 ± 1.22 SD respectively; Table 3). There was substantial rating agreement for CT imaging characteristics (Cohen’s kappa for inter-rater reliability 0.71 and for intra-rater reliability 0.89).

**Table 1.** Demographic info.

Characteristic	All	No dementia	Dementia	*p*-Value
*n*	195	131	64	
Age, mean (SD)	69.65 (12.61)	67.03 (12.95)	75.06 (9.96)	<0.001
Sex (male)	100 (51.5)	71 (54.6)	29 (45.3)	0.286
Education in years, mean (SD)	11.65 (3.36)	11.90 (3.79)	11.07 (2.03)	0.129
Vascular risk factors	32 (16.6)	24 (18.5)	8 (12.7)	0.422
Atrial fibrillation	43 (22.3)	26 (20.0)	17 (27.0)	0.363
Diabetes	20 (10.4)	14 (10.8)	6 ( 9.5)	0.989
Heart failure	115 (59.6)	73 (56.2)	42 (66.7)	0.215
Hypertension	39 (20.2)	23 (17.7)	16 (25.4)	0.290
Dyslipidaemia	61 (31.6)	35 (26.9)	26 (41.3)	0.065
Previous stroke^a^				
Smoking status	52 (27.1)	35 (27.1)	17 (27.0)	0.837
Current	72 (37.5)	50 (38.8)	22 (34.9)	
Former	68 (35.4)	44 (34.1)	24 (38.1)	
Never				
NIHSS total, median (IQR)^b^	2 (0–4)	1 (0–4)	2 (0–5)	0.190^c^
mRS, mean (SD)^d^	1.31 (1.28)	0.99 (1.16)	1.98 (1.27)	<0.001
AMT score median (IQR)^e^	9 (8-10)	9 (8-10)	8 (7-9)	<0.001^c^

SD: standard deviation; NIHSS: National Institutes of Health Stroke Scale; IQR: interquartile range; mRS: modified Rankin Scale; AMT: abbreviated mental test.

*N* (%) unless otherwise indicated. Unpaired *t*-test unless otherwise indicated.

a Self-reported.

b Scores ranging from 0 to 42, lower scores indicating better function.

c Mann-Whitney *U* test.

d Scores ranging from 0 to 4, lower scores indicating better function.

e Scores ranging from 0 to 10, higher scores indicating better function.

**Table 2.** CT imaging parameters.

Characteristic	All	No dementia	Dementia	*p*-Value
*n*	195	131	64	
Stroke type, *n* (%)				
Haemorrhagic	13 (6.7)	8 (6)	5 (8)	0.903
Ischaemic (incl. TIA)	182 (93.3)	123 (94)	59 (92)	
Old infarct, *n* (%)	126 (64.6)	81 (62)	45 (70)	0.316
White matter lesions (WML)^a^				
PVH (0–3)	1.47 (1.07)	1.21 (1.06)	2.00 (0.87)	<0.001
DWML (0–3)	1.19 (1.04)	0.98 (1.00)	1.61 (1.00)	<0.001
Total (0–6)	2.66 (1.92)	2.20 (1.87)	3.61 (1.66)	<0.001
Medial temporal lobe atrophy (MTA)^b^				
Left side (0–4)	2.01 (1.01)	1.76 (0.88)	2.50 (1.07)	<0.001
Right side (0–4)	2.24 (1.04)	2.02 (0.98)	2.70 (1.02)	<0.001
Total (0–8)	4.25 (1.90)	3.78 (1.70)	5.20 (1.95)	<0.001
General atrophy^c^				
Lateral ventricles (0–3)	1.49 (0.42)	1.44 (0.41)	1.62 (0.42)	0.004
IFACC (0–3)	1.79 (0.51)	1.71 (0.51)	1.95 (0.45)	0.002
Sylvian fissures (0–3)	1.82 (0.50)	1.74 (0.50)	1.97 (0.46)	0.003
Occipital sulci (0–3)	1.62 (0.41)	1.56 (0.42)	1.73 (0.39)	0.008
Frontal sulci (0–3)	1.80 (0.42)	1.71 (0.43)	1.98 (0.36)	<0.001
Parietal sulci (0–3)	1.78 (0.45)	1.69 (0.46)	1.95 (0.35)	<0.001
Total atrophy (0–18)	10.28 (2.24)	9.83 (2.30)	11.20 (1.82)	<0.001

TIA: transient ischaemic attack; PVH: periventricular hyperintensity; DWMH: deep white matter lesion; IFACC: interhemispheric fissure anterior to the corpus callosum.

Mean (SD) unless otherwise indicated. Unpaired *t*-test unless otherwise indicated.

a Operationalized by Fazekas scale.

b Operationalized by Scheltens scale.

c Operationalized by Wahlund scale. For all CT imaging parameters do lower scores indicate less degeneration.

**Table 3.** Brain frailty characteristics full cohort.

Characteristic	All	No dementia	Dementia	*p*-Value
*n*	195	131	64	
Individual brain frailty components				
Old infarct	126 (64.6)	81 (61.8)	45 (70.3)	0.316
Severe white matter lesions	104 (53.3)	54 (41.2)	50 (78.1)	<0.001
Severe medial temporal lobe atrophy	134 (69.4)	82 (63.1)	52 (82.5)	0.010
Severe atrophy	154 (79.8)	94 (72.9)	60 (93.8)	0.001
Brain frailty score,^a^ mean (SD)	2.65 (1.19)	2.37 (1.22)	3.24 (0.89)	<0.001
Brain frailty score				<0.001
0	10 ( 5.2)	9 ( 7.0)	1 ( 1.6)	
1	29 (15.2)	27 (21.1)	2 ( 3.2)	
2	32 (16.8)	25 (19.5)	7 (11.1)	
3	66 (34.6)	42 (32.8)	24 (38.1)	
4	54 (28.3)	25 (19.5)	29 (46.0)	

SD: standard deviation.

a Scores ranging from 0 to 4, lower scores indicating less brain frailty.

### Regression analyses

Results of regression analyses are listed in Table 4. Increasing brain frailty was associated with increased risk of dementia (crude hazard ratio (HR), (95% confidence interval (CI)) 1.84 (1.40–2.42) per point increase), even when adjusting for age, sex, history of cardiovascular disease, prestroke mRS, previous stroke and NIHSS (model 4; adjusted hazard ratio (aHR) (95% CI) 1.38 (1.00–1.89) per point increase). Severe WMLs, MTA and atrophy were all associated with increased risk of dementia (crude HR (95% CI): 3.72 (2.06–6.74); 2.37 (1.24–4.54); 4.40 (1.60–12.12) respectively), however, these associations were attenuated for WMLs and atrophy in our adjusted models (model 4; aHR (95% CI) 1.87 (0.94–3.72) and 1.62 (0.53–4.97) respectively).

**Table 4.** Association between increasing brain frailty score and individual (dichotomized) components and dementia.

Variable	HR (95% CI)			
	Model 1; Crude	Model 2^a^	Model 3^b^	Model 4^c^
Brain frailty score^d^ (per point increase)	1.84 (1.40–2.42)	1.63 (1.21–2.20)	1.37 (1.00–1.88)	1.38 (1.00–1.89)
Old infarct	1.35 (0.79–2.31)	1.17 (0.68–2.01)	0.86 (0.49–1.52)	0.86 (0.49–1.52)
Severe WMLs	3.72 (2.06–6.74)	2.64 (1.34–5.17)	1.85 (0.94–3.67)	1.87 (0.94–3.72)
Severe MTA	2.37 (1.24–4.54)	2.42 (1.26–4.64)	2.08 (1.07–4.05)	2.09 (1.07–4.08)
Severe atrophy	4.40 (1.60–12.12)	2.24 (0.71–7.01)	1.62 (0.53–4.96)	1.62 (0.53–4.97)

HR: hazard ratio; CI: confidence interval; WML: white matter lesions; MTA: medial temporal lobe atrophy.

a Adjusted for age and sex.

b Adjusted for age, sex, history of cardiovascular disease, and modified ranking scale (mRS) prestroke.

c Adjusted for age, sex, history of cardiovascular disease, modified ranking scale (mRS) prestroke, previous stroke and NIHSS.

d Scores ranging from 0 to 4, lower scores indicating less brain frailty.

### Sensitivity analyses

**Figure 1.**
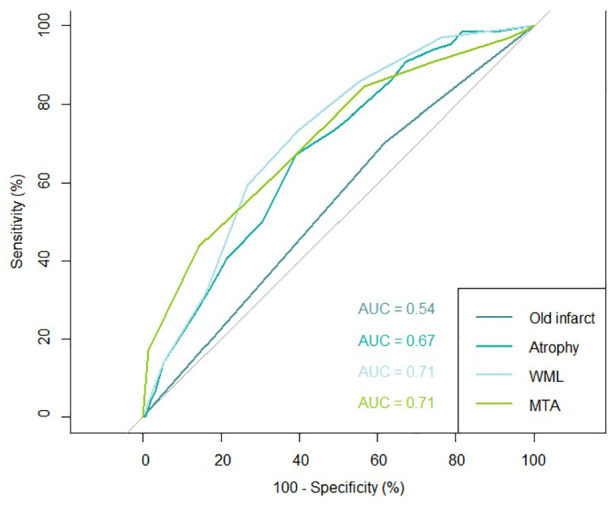
Area under the receiver operating characteristic curve (AUC) for different components brain frailty. WML: white matter lesions; MTA: medial temporal lobe atrophy.

Including death as competing risk in our analyses showed similar outcomes for the association of brain frailty score and its individual components with dementia (Supplemental Tables 1 and 2). Higher brain frailty was associated with higher hazard for dementia with a dose-effect per point increase in the brain frailty score (Supplemental Table 3), although small numbers hamper the interpretation of these analyses. Increasing WML, MTA and atrophy score were all associated with increased risk of dementia (Supplemental Table 4). Individual items of components of the brain frailty score had similar hazard ratios for dementia (Supplemental Table 5). WMLs and MTA had the highest absolute value for area under the curve of all brain frailty components (both 0.71; Figure 1). A posthoc analysis of stroke type (haemorrhagic vs ischaemic) did not significantly alter point estimates for ischaemic stroke (*n* = 183), however, the haemorrhagic stroke group was too small (*n* = 12) to draw any meaningful conclusions (Supplemental Table 6). CT characteristics and brain frailty scores did not differ for participants with pre- and poststroke dementia (*n* = 13 and 53 respectively; Supplemental Table 7).

### Prediction model

The first (backwards selection) model we performed consisted of both clinical and brain frailty components and resulted in a model with MTA, atrophy, age, previous cardiovascular disease, prestroke mRS, NIHSS and AMT-10 at baseline as relevant predictors with good predictive values (*c*-statistic (95% CI) 0.77 (0.71–0.83); Table 5). When excluding cognitive test results, WMLs, MTA, age, previous cardiovascular disease, and prestroke mRS were selected and the discriminative properties only decreased minimally (*c*-statistic (95% CI) 0.74 (0.68–0.80). Clinical parameters only selected age, previous cardiovascular disease and mRS, NIHSS and AMT-10 at baseline and had similar discriminative properties (*c*-statistic (95% CI) 0.76 (0.70–0.82)). For brain frailty parameters only, WMLs, MTA and atrophy were selected as predictors and predictive properties decreased (*c*-statistic (95% CI) 0.69 (0.62–0.75)). Discriminative properties of the backwards selection prediction models were similar to unselected models incorporating all potential predictors (Table 5). Coefficients of the backwards selection models are presented in Supplemental Table 8.

**Table 5.** Discrimination indexes (*c*-statistic) for prediction of dementia incidence.

Variable	Unselected (95% CI)	Backwards selection (95% CI)
Clinical + brain frailty	0.77 (0.71–0.83)	0.77 (0.71–0.83)^a^
Clinical + brain frailty	0.74 (0.68–0.80)	0.74 (0.68–0.80)^b^
Without cognition		
Clinical only	0.76 (0.71–0.82)	0.76 (0.70–0.82)^c^
Clinical only	0.72 (0.66–0.78)	0.72 (0.66–0.78)^d^
Without cognition		
Brain frailty only	0.69 (0.62–0.76)	0.69 (0.62–0.75)^e^

CI: confidence interval.

a Medial temporal lobe atrophy (MTA), atrophy, age, previous cardiovascular disease (CVD), modified ranking scale (mRS), National Institutes of Health Stroke Scale (NIHSS) and Abbreviated Mental Test (AMT-10).

b White matter lesions (WMLs), MTA, age, CVD, mRS.

c Age, CVD, mRS, NIHSS and AMT-10.

d Age, CVD and mRS.

e WMLs, MTA and atrophy.

## Discussion

In this cohort of stroke survivors, we reported that a higher brain frailty score on acute stroke CT imaging was associated with higher risk of dementia. The presence of severe WMLs, MTA and atrophy were all independently associated with a higher risk of dementia, but this effect appeared to be modulated through other comorbidities such as cardiovascular disease and prestroke mRS. Combining clinical parameters and brain frailty components led to a moderate discriminative prediction model for dementia, but had limited added value over clinical parameters alone when cognitive testing was included. However, as CT imaging is standard care in the acute stroke work-up and especially cognitive clinical testing may pose challenges in stroke survivors, CT could be more readily accessible for certain patients.

### Comparison with previous literature

Previously, perfusion CT imaging and MRI scans have been demonstrated to be usable as prognostic tool for dementia in unselected populations.^24,25^ Ultimately these CT perfusion and MRI markers are a reflection of underlying cerebrovascular pathology which contribute to both acute stroke occurrence and long-term cognitive decline. In line with our results, white matter lesions on MRI, irrespective of location, have been associated with worse functional outcomes and more cognitive decline in both unselected and stroke populations.^24,26,27^ A recent systematic review reported that combined cerebral atrophy, presence and severity of white matter hyperintensities (WMH) and presence of cerebral micro-bleeds on acute stroke MRI was associated with a higher risk of post-stroke cognitive decline and dementia.^6^ This study added that pre-existing neurodegeneration was more related to cognitive outcome than acute stroke related lesions. Another study found comparable outcomes in lacunar stroke survivors exhibiting higher levels of brain frailty on acute CT imaging, which correlated with less favourable outcomes, including worse clinical performance and cognitive scores.^28^ Our study adds to these findings by demonstrating that acute CT imaging can also be utilized in unselected stroke populations (lacunar, cortical and haemorrhagic) and can help to detect increased risk of dementia. In our cohort, only severe atrophy and MTA were identified as relevant predictors of dementia in the backward selection model, indicating limited added value of the composite brain frailty score. However, this finding should be interpreted with caution given the small cohort size and the potential associations suggested by point estimates for all brain frailty components.

Although the models with either cognitive testing or CT imaging had similar associations with dementia, the routine use of CT in practice, and the limited application of acute stroke cognitive screening, supports the value of using the CT brain frailty measure. The CT measure may have particular utility when cognitive testing is problematic due to symptoms as aphasia or interfering morbidity.

Previously, we found that evaluating brain frailty using routinely obtained CT scan images is an effective way to determine a person’s brain resilience, and aligns with clinical definitions of physical frailty.^29^ In this study, we reported that clinical parameters had good discriminative properties for predicting incident dementia and that adding CT imaging parameters had limited added value. These findings are in line with a previous cohort study that reported that prior stroke, stroke severity, and baseline cognitive and functional status were all independently associated with post-stroke incident dementia.^30^ A systematic review combining three cohorts of participants with asymptomatic or symptomatic WMHs reported similar discriminative properties to our study when combining clinical and MRI characteristics, implying that CT imaging does not have to be inferior to MRI imaging for this purpose.^31^ This systematic review did report a marginal improvement in predicting dementia by adding markers for small vessel disease (SVD) to clinical parameters (AUC 0.76–0.81), however, in this study, baseline functioning of cognitive performance was not included.^31^ A considerable limitation for these studies and the current study is that none of these risk models have been externally validated, posing a significant challenge in clinical practice due to the uncertainty surrounding their reliability and generalizability across diverse patient populations.^32^

### Strengths and limitations

This study has several strengths. Our cohort was formed using a very inclusive approach, ensuring a representative sample reflective of real-world acute stroke populations. We ensured blinding and double scoring to minimize risk of bias and reported a very high reproducibility of CT scan rating. Additionally, even though 18-month cognitive test data were unavailable for some participants, the use of the earlier cognitive test data and additional electronic health record data from primary and secondary care still ensured thorough and complete follow-up of participants. We established comprehensive dementia adjudication procedures, enhancing the reliability and validity of our findings. There are also several limitations. For clinical applicability, we used readily available neuroimaging scoring lists that could be applied by clinicians, however ordinal measures can lack precision and advanced computational techniques, such as volumetric analyses of factors as atrophy and white matter burden, could yield better predictive properties for dementia. This straightforward approach, however, does allow for broad implementation across a wide range of health care settings, including LMICs. We also observed a slightly higher dementia rate than in previous literature. This could be due to the dementia classification of both probable and possible dementia cases, which may have led to some patients being classified as having dementia when mild cognitive impairment (MCI) might have been more appropriate. Additionally, the regular follow-up and detailed assessments in this study cohort may have increased the detection rate of dementia compared to typical community settings. Moreover, our cohort included recurrent stroke and placed no restrictions on pre-stroke morbidity or function. Furthermore, we were unable to internally validate our findings because of the limited power within our sample. Lastly, we were unable to provide insight in the trajectory of cognitive impairment following stroke, as our cognitive outcome measurements were too heterogeneous to allow for a longitudinal analysis.

### Future implications and directions

CT imaging remains pivotal in the initial evaluation of stroke patients due to its widespread accessibility, rapid acquisition time, and cost-effectiveness. Validating CT scans as prognostic tools, particularly in regions where MRI scans are not widely available, might help clinicians to harness readily available imaging data to inform conversations around prognosis and prevention of (post-stroke) dementia. A better understanding of the risk of dementia would help guide early counselling and advance care planning, and might ultimately improve quality of (early) dementia care. In research, a non-invasive means of selecting out the highest-risk individuals will increase trial efficiency when specifically aiming to reduce the risk of dementia. Such a prognostic biomarker may reduce required sample sizes for clinical trials by increasing event rates, leading to more efficient trials, which are less costly and have a higher chance of success. A cohort study demonstrated that selecting participants with a high SVD score (similar to high brain frailty) had the ability to reduce clinical trial sample sizes up to 57% when assuming a 10% treatment effect and looking at dementia as primary outcome.^31^ In the future, CT imaging might allow for trials of disease modifying therapeutics before clinically apparent, and as yet irreversible, cognitive impairment occurs by identifying people at high risk of dementia.^33^ Additionally, acute imaging artificial intelligence could potentially enable automatic calculations of brain frailty in stroke imaging, similar to the advancements currently being made for ASPECTS scores and CT perfusion analyses.^34,35^

The findings of this study underscore the potential of CT imaging in acute stroke as a valuable adjunctive tool for enhancing our understanding of dementia and refining clinical trial design. We demonstrated that the application of simple, readily available rating scales for stroke CT scans and additional clinical information may help to better inform about dementia risk, particularly when cognitive testing is impractical because of interfering morbidity. This suggests that CT imaging, despite its conventional role in acute stroke diagnosis, may serve as an informative predictor of long-term cognitive outcomes. External validation of these findings is needed to help establish robust predictive models for informed patient management strategies.

## Supplemental Material

sj-docx-1-eso-10.1177_23969873251325076 – Supplemental material for Prediction of dementia using CT imaging in stroke (PRODUCTS) Supplemental material, sj-docx-1-eso-10.1177_23969873251325076 for Prediction of dementia using CT imaging in stroke (PRODUCTS) by Melanie Hafdi, Martin Taylor-Rowan, Bogna Drozdowska, Emma Elliott, Lucy McGuire, Edo Richard and Terence J Quinn in European Stroke Journal
