# Supplementary material for: Prediction of dementia using CT imaging in stroke (PRODUCTS)
Source: Eur Stroke J. 2025 Mar 13;10(3):978–87. doi: 10.1177/23969873251325076 (PMC11907507; doi:10.1177/23969873251325076)
Supplement: sj-docx-1-eso-10.1177_23969873251325076 – Supplemental material for Prediction of dementia using CT imaging in stroke (PRODUCTS) [file sj-docx-1-eso-10.1177_23969873251325076.docx]

**Supplement tables**

**Supplement table 1**. Association between individual components of brain frailty (dichotomized) and dementia using Fine - Gray subdistribution hazard model

|  | HR (95% CI) | | | |
| --- | --- | --- | --- | --- |
|  | *Model 1; Crude* | *Model 2 ^1^* | *Model 3 ^2^* | *Model 4 ^3^* |
| Brain frailty score ^4^  (per point increase) | 1.81 (1.39-2.39) | 1.61 (1.19-2.16) | 1.40 (1.02-1.92) | 1.41 (1.02-1.94) |
| Old infarct | 1.35 (0.79-2.31) | 1.19 (0.69-2.04) | 0.89 (0.51-1.57) | 0.89 (0.51-1.58) |
| Severe WMLs | 3.72 (2.06-6.74) | 2.67 (1.37-5.23) | 1.94 (0.98-3.83) | 1.98 (1.00-3.94) |
| Severe MTA | 2.15 (1.15-4.03) | 2.20 (1.18-4.14) | 2.10 (1.08-4.09) | 2.09 (1.07-4.08) |
| Severe atrophy | 4.48 (1.63-12.35) | 2.28 (0.73-7.11) | 1.72 (0.56-5.28) | 1.73 (0.56-5.33) |

*Abbreviations:* HR; hazard ratio, CI; confidence interval, WML; white matter laesions, MTA; medial temporal lobe atrophy. ^1^ Adjusted for age and sex. ^2^ Adjusted for age, sex, history of cardiovascular disease, and mRS prestroke. ^3^ Adjusted for age, sex, history of cardiovascular disease, modified ranking scale (mRS) prestroke, previous stroke and NIHSS ^4^ Scores ranging from 0-4, lower scores indicating less brain frailty

**Supplement table 2**. Association between individual components brain frailty (full score) and dementia (adjusted) using Fine - Gray subdistribution hazard model

|  | HR (95% CI) | | | |
| --- | --- | --- | --- | --- |
|  | *Model 1; Crude* | *Model 2 ^1^* | *Model 3 ^2^* | *Model 4 ^3^* |
| Brain frailty  (per point increase) | 1.81 (1.39-2.39) | 1.61 (1.19-2.16) | 1.40 (1.02-1.92) | 1.41 (1.02-1.94) |
| Infarct | 1.35 (0.79-2.31) | 1.19 (0.69-2.04) | 0.89 (0.51-1.57) | 0.89 (0.51-1.58) |
| WML (0-6) | 1.35 (1.18-1.54) | 1.25 (1.07-1.46) | 1.13 (0.96-1.33) | 1.14 (0.97-1.34) |
| MTA (0-8) | 1.42 (1.24-1.63) | 1.32 (1.14-1.53) | 1.23 (1.06-1.44) | 1.24 (1.05-1.45) |
| Atrophy (0-18) | 1.27 (1.21-1.43) | 1.14 (0.97-1.34) | 1.04 (0.88-1.23) | 1.04 (0.88-1.23) |

*Abbreviations:* HR; hazard ratio, CI; confidence interval, WML; white matter laesions, MTA; medial temporal lobe atrophy. ^1^ Adjusted for age and sex. ^2^ Adjusted for age, sex, history of cardiovascular disease, and mRS prestroke. ^3^ Adjusted for age, sex, history of cardiovascular disease, modified ranking scale (mRS) prestroke, previous stroke and NIHSS

**Supplement table 3**. Association between brain frailty score (dichotomized) and dementia

|  | Dementia (n/N) | HR (95% CI) | p-value |
| --- | --- | --- | --- |
| Brain frailty score  (0 or 1; reference) | 3/39 |  |  |
| Brain frailty ≥2 | 60/152 | 6.02 (1.89-19.21) | 0.002 |
| Brain frailty ≥3 | 53/120 | 6.97 (2.17-22.31) | 0.001 |
| Brain frailty 4 | 29/54 | 9.05 (2.75-29.77) | 0.002 |

*Abbreviations:* HR; hazard ratio, CI; confidence interval

**Supplement table 4**. Association between individual components brain frailty (full score) and dementia (adjusted)

|  | HR (95% CI) | | |  |
| --- | --- | --- | --- | --- |
|  | *Model 1; Crude* | *Model 2 ^1^* | *Model 3 ^2^* | *Model 4 ^3^* |
| Brain frailty (per point increase) | 1.84 (1.40-2.42) | 1.63 (1.21-2.20) | 1.37 (1.00-1.88) | 1.38 (1.00-1.89) |
| Infarct | 1.35 (0.79-2.31) | 1.17 (0.68-2.01) | 0.86 (0.49-1.52) | 0.86 (0.49-1.52) |
| WML (0-6) | 1.35 (1.18-1.54) | 1.24 (1.06-1.45) | 1.12 (0.95-1.32) | 1.13 (0.96-1.33) |
| MTA (0-8) | 1.42 (1.24-1.63) | 1.33 (1.15-1.55) | 1.22 (1.05-1.43) | 1.23 (1.05-1.44) |
| Atrophy (0-18) | 1.27 (1.12-1.43) | 1.16 (0.98-1.36) | 1.03 (0.87-1.21) | 1.03 (0.87-1.21) |

*Abbreviations:* HR; hazard ratio, CI; confidence interval, WML; white matter laesions, MTA; medial temporal lobe atrophy.
^1^ Adjusted for age and sex. ^2^ Adjusted for age, sex, history of cardiovascular disease, and mRS prestroke. ^3^ Adjusted for age, sex, history of cardiovascular disease, modified ranking scale (mRS) prestroke, previous stroke and NIHSS

|  | HR (95% CI) |
| --- | --- |
| Old infarct, n (%) | 1.35 (0.79-2.31) |
| White matter lesions (0-6) ^1^  PVL (0-3)  DWML (0-3) | 1.35 (1.18-1.54)  1.75 (1.37-2.23)  1.57 (1.22-1.94) |
| Medial temporal lobe atrophy (0-8) ^2^  Left side (0-4)  Right side (0-4) | 1.42 (1.24-1.63)  1.84 (1.44-2.35)  1.79 (1.38-2.31) |
| General atrophy (0-18) ^3^  Lateral ventricles (0-3)  IFACC (0-3)  Sylvian fissures (0-3)  Occipital sulci (0-3)  Frontal sulci (0-3)  Parietal sulci (0-3) | 1.27 (1.12-1.43)  2.28 (1.29-4.04)  2.11 (1.28-3.47)  2.09 (1.25-3.51)  2.19 (1.20-4.01)  3.73 (1.94-7.18)  3.37 (1.78-6.39) |
| Weighed score (total) | 1.18 (1.09-1.27) |

**Supplement table 5**. Individual components of scales and dementia

*Abbreviations*: PVL; periventricular lesions, DWML; deep white matter lesions, IFACC; interhemispheric fissure anterior to the corpus callosum

**Supplement table 6**. Association between individual components brain frailty and dementia per stroke type

|  | HR (95% CI) | | |
| --- | --- | --- | --- |
|  | *All participants (n=195)* | *Ischemic*  *(n=183)* | *Haemorrhagic*  *(n=12)* |
| Brain frailty score ^3^  (per point increase) | 1.84 (1.40-2.42) | 1.96 (1.46-2.63) | 1.12 (0.51-2.47) |
| Old infarct | 1.35 (0.79-2.31) | 1.55 (0.87-2.79) | 0.423 (0.05-3.81) |
| Severe WMLs | 3.72 (2.06-6.74) | 4.02 (2.13-7.58) | 2.10 (0.35-12.6) |
| Severe MTA | 2.37 (1.24-4.54) | 2.36 (1.22-4.55) | Not available ^1^ |
| Severe atrophy | 4.40 (1.60-12.12) | 5.28 (1.65-16.9) | 1.95 (0.22-17.50) |
|  | | | |

*Abbreviations:* HR; hazard ratio, CI; confidence interval, WML; white matter laesions, MTA; medial temporal lobe atrophy
^1^ All participants with haemorrhagic stroke had severe MTA

**Supplement table 7**. CT imaging parameters for pre- and poststroke dementia

|  | Dementia | Prestroke dementia | Poststroke dementia | p-value |
| --- | --- | --- | --- | --- |
| n | 64 | 13 | 51 |  |
| Individual brain frailty components  Old infarct   Severe white matter lesions  Severe medial temporal lobe atrophy  Severe atrophy | 45 (70.3)  50 (78.1)  52 (82.5)  60 (93.8) | 10 (76.9)  9 (69.2)  10 (83.3)  11 (84.6) | 35 (68.6)  41 (80.4)  42 (82.4)  49 (96.1) | 0.807  0.622  1.000  0.378 |
| Brain frailty score, mean (SD) | 3.24 (0.89) | 3.08 (1.38) | 3.27 (0.75) | 0.509 |
| Brain frailty score  0  1  2  3  4 | 1 ( 1.6)  2 ( 3.2)  7 (11.1)  24 (38.1)  29 (46.0) | 1 (8.3)  1 (8.3)  1 (8.3)  2 (16.7)  7 (58.3) | 0 (0.0)  1 (2.0)  6 (11.8)  22 (43.1)  22 (43.1) | 0.096 |

*Abbreviations*: SD; standard deviation

**Supplement table 8**. Coefficients of backwards selection prediction models

|  | Coefficient |
| --- | --- |
| Clinical + brain fraity  Severe MTA  Severe atrophy  Age  CVD  mRS  NIHSS  AMT-10 | 0.55  0.78  0.03  0.56  0.40  -0.06  -0.34 |
| Clinical + brain frailty  *without cognition*  Severe WMLs  Severe MTA  Age  CVD  mRS | 0.66  0.75  0.03  0.42  0.33 |
| Clinical only  Age  CVD  mRS  NIHSS  AMT-10 | 0.40  0.46  0.43  -0.06  -0.34 |
| Brain frailty only  Severe WMLs  Severe MTA  Severe atrophy | 1.11  0.75  0.79 |

*Abbreviations*: MTA; medial temporal atrophy, CVD; cardiovascular disease, mRS; modified Rankin Scale, NIHSS; National Institutes of Health Stroke Scale, AMT-10; Abbreviated Mental Test (10-item version), WMLs; white matter lesions.
